# Views of Women and Health Professionals on mHealth Lifestyle Interventions in Pregnancy: A Qualitative Investigation

**DOI:** 10.2196/mhealth.4869

**Published:** 2015-10-28

**Authors:** Jane C Willcox, Paige van der Pligt, Kylie Ball, Shelley A Wilkinson, Martha Lappas, Elizabeth A McCarthy, Karen J Campbell

**Affiliations:** ^1^Centre for Nutrition and Physical Activity ResearchSchool of Nutrition and Exercise SciencesDeakin UniversityBurwoodAustralia; ^2^Centre for Nutrition and Physical Activity ReserchSchool of Nutrition and Exercise SciencesDeakin UniversityBurwoodAustralia; ^3^Mater Research InstituteUniversity of QueenslandSouth BrisbaneAustralia; ^4^Mater Mothers HospitalBrisbaneAustralia; ^5^Department of Obstetrics and GynaecologyUniversity of MelbourneMercy Hospital for WomenHeidelbergAustralia

**Keywords:** pregnancy, attitude, qualitative research, mHealth

## Abstract

**Background:**

Evidence suggests that women are failing to meet guidelines for nutrition, physical activity, and weight gain during pregnancy. Interventions to promote a healthy lifestyle in pregnancy demonstrate mixed results and many are time and resource intensive. mHealth-delivered interventions offer an opportunity to provide trusted source information in a timely and cost-effective manner. Studies regarding women’s and health professionals’ views of mHealth in antenatal care are limited.

**Objective:**

This study aimed to explore women’s and health professionals’ views regarding mHealth information sources and interventions to assist women to eat well, be physically active, and gain healthy amounts of weight in pregnancy.

**Methods:**

A descriptive qualitative research approach employed focus groups and in-depth interviews with 15 pregnant or postpartum women and 12 in-depth interviews with health professionals including two from each category: obstetricians, general practitioners, midwives, dietitians, physiotherapists, and community pharmacists. All interviews were transcribed verbatim and thematically analyzed.

**Results:**

Women uniformly embraced the concept of mHealth information sources and interventions in antenatal care and saw them as central to information acquisition and ideally incorporated into future antenatal care processes. Health professionals exhibited varied views perceiving mHealth as an inevitable, often parallel, service rather than one integrated into the care model. Four key themes emerged: engagement, risk perception, responsibility, and functionality. Women saw their ability to access mHealth elements as a way to self-manage or control information acquisition that was unavailable in traditional care models and information sources. The emergence of technology was perceived by some health professionals to have shifted control of information from trusted sources, such as health professionals and health organizations, to nontrusted sources. Some health professionals were concerned about the medicolegal risks of mHealth (incorrect or harmful information and privacy concerns), while others acknowledged that mHealth was feasible if inherent risks were addressed. Across both groups, there was uncertainty as to who should be responsible for ensuring high-quality mHealth. The absence of a key pregnancy or women’s advocacy group, lack of health funds for technologies, and the perceived inability of maternity hospitals to embrace technology were seen to be key barriers to provision. Women consistently identified the functionality of mHealth as adding value to antenatal care models. For some health professionals, lack of familiarity with and fear of mHealth limited their engagement with and comprehension of the capacity of new technologies to support antenatal care.

**Conclusions:**

Women exhibited positive views regarding mHealth for the promotion of a healthy lifestyle in antenatal care. Conversely, health professionals expressed a much wider variation in attitudes and were more able to identify potential risks and barriers to development and implementation. This study contributes to the understanding of the opportunities and challenges in developing mHealth lifestyle interventions in antenatal care.

## Introduction

Nutrition, physical activity and gestational weight gain (GWG) during pregnancy can impact both a woman’s and offspring’s short and long-term health [1-4]. Pregnant women typically value lifestyle advice [5] and are receptive to opportunistic health promotion interventions during the antenatal period [6]. Despite this, suboptimal physical activity, diet, and GWG are commonly reported [7,8]. Hospital and community-based resources to assist pregnant women achieve better nutrition and physical activity and optimize GWG may be limited [9]. In research settings, there is a growing body of evidence to support the use of interventions promoting diet and exercise, or both, to reduce excessive GWG in pregnancy [10,11], but given many are time and resource intensive, scalability is limited. Novel and sustainable ways to extend the reach to all women are required.

The methods by which women acquire lifestyle information in pregnancy are changing with increasingly accessible health information in digital format [12], while increasing documentation requirements are leading to shorter patient-provider interactions [13]. Lagan and colleagues surveyed 613 pregnant women from 24 countries to explore Internet use and its effect on their health decision-making [12]. Nearly 94% (575/613) of women used the Internet in addition to health professionals to get pregnancy information, and 83% used it to influence their pregnancy decision-making. Arguably, health services have been slow to develop information technology to satisfy the requirements of consumers.

Mobile phones have been widely adopted among all demographic groups and are increasingly used as a platform for delivering programs to support the achievement of health objectives, commonly referred to as mHealth [14]. mHealth offers an opportunity to provide trusted source information and interventions incorporating behavioral change practices through a low-cost, easy access method [14,15]. mHealth utilities used in health interventions have included text message or short message service, apps, video messaging, handheld computers, voice calls, and audio packages [15]. Systematic reviews of interventions using mHealth have found increased adherence to antiretroviral therapy, smoking cessation, engagement in physical activity, and weight loss [15-17].

A recent systematic review of technology supporting dietary and lifestyle interventions in pregnant women found four protocols and three completed studies using telephone, text messaging, video, Internet, and apps [18]. The authors concluded that mHealth interventions hold promise for interactive, practical, accessible, and instantaneous support but that there was a paucity of data on mHealth effectiveness for pregnant women. They recommended further randomized controlled trials supporting health behavior change in real-life clinical settings.

Understanding women’s and health professionals’ views and attitudes regarding the promotion of healthy nutrition, physical activity, and GWG with mHealth is crucial to assist in the development of practical, time-efficient, and cost-effective ways to promote healthy lifestyles in pregnancy and plan mHealth evaluations [17-19]. A number of papers have emphasized the importance of understanding and incorporating stakeholders’ views into design and evaluation [19-22]. In Heron and Smyth’s review of mobile technology in psychosocial and health behavior treatments, for example, the authors suggest that interventions need to be more sensitive to the individual characteristics and needs of stakeholders [17]. They argue that incorporating end user and provider feedback into design, implementation, and evaluation will facilitate feasibility and acceptability of interventions.

A small number of recent studies have examined pregnant women’s and midwives’ views of websites and apps or text messaging to promote healthy nutrition, physical activity, and GWG in pregnancy [20-22]. These studies demonstrated an interest among women [20-22] and midwives [20] for text messages [20,21], apps, and websites [22]. Soltani and colleagues used focus groups of women and midwives to understand their perspectives on the design of text messaging support for maternal obesity services [20]. Three main constructs emerged: benefits, risks and limitations, and service delivery of a text message program. Further, participants suggested the use of technology platforms such as Web-based services in addition to text messaging for weight management in pregnancy. The authors used these results to construct a small pilot text messaging intervention [23]. We are unaware of studies exploring other health professional stakeholder views, for example, those of dietitians, physiotherapists, general practitioners, pharmacists, and obstetricians in addition to midwives. Opportunity exists to broaden understanding of views on how mHealth and its range of technology platforms could be used to promote healthy lifestyles in antenatal care.

This study aimed to explore women’s and health professionals’ views regarding mHealth information sources and interventions to assist women to eat well, be physically active, and gain healthy amounts of weight in pregnancy.

## Methods

### Design and Ethics

A qualitative, descriptive research methodology [24,25] using face-to-face semistructured interviews and focus groups was employed to obtain in-depth data from consenting participants. Ethics approval was obtained from Deakin University (2013-213) and Mercy Hospital for Women (R13/27) Human Research Ethics Committees.

### Study Participants

The study recruited pregnant or postpartum women from antenatal clinics in a tertiary level maternity hospital in Victoria, Australia, and proceeded to increase the number of participants using snowball sampling [26]. To ensure that views from a broad range of health professionals were included, two each of the following categories were purposively sampled [27] from both Victoria and Queensland: obstetricians, general practitioners, midwives, dietitians, physiotherapists, and community pharmacists. The sample size was limited by budget and informed by similar studies with midwives [20]. Women and health professionals were invited to participate in interviews or focus groups via written or face-to-face invitation.

### Data Collection

Focus groups and face-to-face interviews with women and face-to-face interviews with health professionals were conducted by JW and PvdP using standardized interview guides (see Multimedia Appendix 1). The interview only methodology with health professionals was chosen for pragmatic time-related reasons. The lack of focus group interaction among health professionals, potentially yielding less in-depth interactive information, is acknowledged as a potential limitation.

The content of the interview guides was informed by the literature, including correlates of healthy pregnancy lifestyles [28], predictors of health behaviors [29], successful elements of pregnancy lifestyle interventions [10], and mHealth interventions [15] and the uses and gratification theory, a theory of media usage [30]. Semistructured and structured questions to elicit women’s and health professionals’ views, attitudes, and practices around mobile phones and mHealth, as well as their thoughts on optimal interventions, were investigated during the interviews and focus groups. A visual diagram including text messaging, social networking, video messaging, websites, print media, and health professional interaction was used to provide a guide to direct the discussion (Multimedia Appendix 1).

Common themes explored included mHealth’s suitability and viability to provide healthy lifestyle advice and support to pregnant women, mHealth inclusion in antenatal care, use and suitability of mHealth elements, barriers and facilitators to mHealth development and implementation, and inclusions and exclusions in a program to support healthy lifestyle advice. In addition, sociodemographic characteristics of all participants were collected, as well as data on women’s parity and the health professional’s role and length of employment in their profession. The interviews were digitally recorded with the consent of the participants and transcribed verbatim.

### Data Analysis

Data immersion, coding, category creation, and thematic analysis were used to find patterns of meaning across data sets [31,32]. The researchers JW and PvdP used an inductive approach to derive themes through interpretations of the raw data [33]. Coding categories and subcategories were allocated by the two independent researchers, and the congruence was assessed and found to be good. Discrepancies were discussed and resolved to reduce researcher bias during the thematic development phase. Both researchers agreed on the final category system and accepted it as being representative of the data.

## Results

### Participants

Fifteen women participated in either one of two focus groups (n=7) or an interview (n=8). Three additional women declined participation. Eleven of the women were pregnant and four postpartum (5-18 months). The mean age of the women in the study was 31.5 years, two were born outside Australia, nine were first-time mothers, and all but one owned a mobile phone. Two health professionals from each group—obstetricians, general practitioners, midwives, dietitians, physiotherapists, and community pharmacists—participated and averaged 8.3 years (range 4-27 years) practice in their current professions; all owned mobile phones. All health professionals approached participated in the study. Construct saturation, representation of content area, was reached with the women at 13 participants but interviews continued to confirm saturation. Similar saturation was reached for health professionals at 12 participants. The health professional quotes have not been differentiated due to the possibility of interviewee identification and thus breach of anonymity.

### Emergent Themes

#### Overall

Women uniformly embraced the concept of mHealth-based interventions in antenatal care and saw them as central to information acquisition and in future antenatal care processes. The health professionals exhibited a wider variation in views towards mHealth in antenatal care. They saw it as an inevitable but often parallel service rather than integrated into the care model. Four key themes (Textbox 1) emerged from both women’s and health professionals’ data: engagement, risk perception, responsibility, and functionality.

**Themes and subthemes from interviews and focus groups regarding antenatal mHealth programs.**

Engagement:

- Good access point
- Women engaged and technically proficient
- Health professionals less engaged and less technically proficient
- Allows wide reach

Perceived risk:

- Familiarity with technology reduced fear of risk for women
- Potential for causing harm and stress for women
- Potential for harm to professional integrity of health professionals and organizations
- Shifting control of information

Responsibility:

- Responsibility of government, health services, and health professionals
- Barriers included lack of advocacy, funds, information technology know-how, and commercial application

Functionality:

- Role in antenatal care
- Multiple technology elements
- Optimize user engagement and experience
- Evidence-based, practical content and delivery

#### Theme 1: Engagement and Access

Mobile phones were uniformly seen by both groups (health professionals [HP] and women [W]) to be a good access point for intervention programs with pregnant women, including at-risk women who may not attend antenatal visits.

I like to receive things on my phone, I like regular updates.

W8, focus group

Every person that comes in here has either a smart phone or an iPhone. It doesn’t matter what nationality or how poor they are, they’ve all got (one) and the most up-to-date one.… It’s capturing the people that miss out…the marginalized people that always slip through the system, you can catch them.

HP6

All women and some health professionals acknowledged that women were already engaged with health-related technology and wanted mHealth products to use. Some women considered their ability to access mHealth and related technology as providing an important adjunct to their traditional medical care. Future development of mHealth programs was seen to augment this. There was an acknowledgement from four health professionals that women were potentially more technically savvy than health professionals.

An [mHealth] concept would fill a gap since no one mentioned nutrition or anything barely to me in the medical profession. They seem to think it’s not their domain…so it would be good to fill the gap.

W9, interview

Women are incredibly technical…they will be keen or interested because I see it all the time. They say to me, “Where shall I go, what should I eat, what websites or apps?”

HP1

While phones or mobile devices were acknowledged to be good methods to communicate with women, some health professionals’ dislike of or unfamiliarity with technology limited engagement and understanding of the capacity of new technologies to support care.

a lot of my [health professional] cohorts wouldn’t be as engaged with all of that.

HP4

There was concern expressed by some health professionals for the minority of women who may not have mobile or Internet access and the need to provide alternatives.

I think most people nowadays have access. There’ll always be a small group of people that are disadvantaged and so you need a print back-up but I do think it’s the way of the world now.

HP12

The scalability of an mHealth product was seen to be important for the wide engagement of women. Mobile phones or tablets functions were seen to reach a wider audience than traditional methods.

It should be able to be rolled out to the whole country.

W12, interview

Where does that go in the long term, and how can [mHealth] be scaled and controlled to be a beneficial way of providing support to women?

HP4

#### Theme 2: Perceived Risk

Many women appreciated the risks involved in accessing technology and in the development of an mHealth model. Many saw themselves as having the capacity to evaluate and select information to suit their situation and the ability to sift out noncredible information.

I make sure I know it’s a trusted source. I know the trusted [sources] so I just kind of go off them if there is something I need to know.

W8, focus group

Many health professionals and two women were concerned about the medicolegal risks of technology. These risks were divided into two categories: harm to women (incorrect or harmful information, privacy concerns) and harm to the personal or professional integrity of health professionals and health organizations (intellectual property, privacy, and legitimacy concerns). This group was also worried that an intervention program may cause harm or stress to women. Two health professionals expressed unease about messages being shared out of context and misinterpreted by other pregnant women.

As a health care professional, I’m just mindful that if there was a video of me up there talking, if that was taken out of context or shared with another person where that information was not appropriate, that’s a concern to me.

HP9

This is a new era…we can’t tell them which technologies [websites, apps] are bad [and good]…because we’ll get sued.

HP1

A small number of health professionals and women were concerned that not meeting an mHealth program’s expectations may be stressful for women. One health professional commented on the changing needs of women during pregnancy, where advice may change depending on a medical situation or potential for harm.

Some women might find it [an mHealth program promoting a healthy lifestyle] anxiety provoking.

W9, interview

If you get someone that’s early in their pregnancy, but then they develop a medical problem or say pelvic girdle pain, I’m thinking it wouldn’t be appropriate to tell them to take the stairs, because that’ll actually make their pain worse. So do you have the ability to pull them out or change it?

HP9

Four health professionals discussed the perceived detrimental effects to the health professional–patient relationship with the advent of health-related technology and the shifting of control of information from trusted to nontrusted, including commercial, sources. Future mHealth programs were seen by some to potentially continue the trend of information contradiction and control. Three health professionals questioned mHealth programs taking control or legitimacy away from on the ground health professionals, but others saw future evidence-based mHealth programs being able to wrest this control back. Further, two health professionals expressed concerns about the risk of losing control of information provision with commercialization of lifestyle education mHealth programs and questioned who would ultimately benefit.

The Internet [and other technologies] have more legitimacy than [HPs] or written info.

HP6

You’re encroaching on dietitians’ territory here…a dietitian might get upset if somebody else was doing it.

HP3

#### Theme 3: Responsibility for mHealth

Across both groups there was uncertainty as to who should be responsible for ensuring high-quality interventions via mHealth. Many women and health professionals suggested that maternity hospitals, general practitioners, or government health departments should take responsibility for mHealth in pregnancy to ensure legitimacy of the information provided and accessed.

I think hospitals need to get the right information out there so that they’re not having all these women find bits and pieces everywhere else.

HP10

I think it should be introduced at the first antenatal visit [by maternity hospitals], with health professionals recommending and advising on it.

W13, interview

The absence of a key pregnancy or women’s advocacy group, lack of health funds for technologies, and the perceived inability of maternity hospitals and governments to embrace technology were seen to be key barriers to provision. The lack of commercial apps for such a program was seen to also be an obstacle to gain funding.

Health in Australia has been slow to pick up the [technologies] and partly because they’re a little bit scared.… All the [technologies] that the public knows have accessibility in the public realm and are visible, say the National Heart Foundation. Pregnancy doesn’t have that.

HP11

I totally agree with [mHealth], but I think it comes down to the concept of funding—who’s going to fund it? …I have a lack of confidence in government doing it based on past experience with IT.

HP5

#### Theme 4: Functionality

Women consistently identified the functionality of technology as adding value to antenatal care models. All women were able to comment on different elements and interactions they desired in a mHealth program. While some health professionals were able to discuss elements of mHealth and service delivery concepts, for others unfamiliarity with technology and fear of loss of control of the information provision limited their engagement with and comprehension of the capacity of new technologies.

### Role in Antenatal Care

There was a distinct difference between the two groups as to whether mHealth could be integrated into or should augment traditional antenatal care. Women’s use of current technologies allowed them to envisage mHealth inclusion in traditional care.

[mHealth] should be included in your first visit to the midwife at the hospital. Just take a consent…and sign her up.

W2, focus group

being supported by the technology helps, because you've got that personal interaction to start explaining it and getting people kind of aware and engaged and know what's going on before [using] technology.

W12, interview

Some health professionals viewed mHealth as adding value to the consult with some suggesting that it could help direct women away from the nontrusted sources of information. Two health professionals expressed views about mHealth not having anything to offer antenatal care in comparison to face-to-face interactions

I could see myself saying that there’s this brilliant thing that will help you coordinate your diet and exercise for the pregnancy.… I don’t have the time and resources to go into that and most people are savvy enough.

HP11

They want to be listened to, they want to know that they have been heard. And we can’t do that from a text message or an app. And that’s what they like about coming in and getting that face to face.

HP1

Many health professionals commented that an mHealth program needed to be introduced by health professionals and adjunct to health professional care. Health professionals viewed a program to be optimized by personal connection and written information to guide the user. Some women perceived a benefit in this type of combined approach but many were concerned by the time that this would take and viewed their technological abilities as adequate to adopt a program without instruction.

You have to create a connection with them before they start using it.

HP1

The face-to-face midwife, I don’t know, it would depend on…how much it’s taking out of our time.

W8, focus group

### Multiple Technology Elements

#### Integration of Technology Elements

Both groups articulated that the individual requirements of women would be best served by programs or interventions that integrated multiple technology elements. This was also seen to serve the needs of different learning styles.

I like the concept of a one-stop shop [with different platforms]…across different mediums. So it’s for every woman—every pregnant woman can gain something from this.

W15, interview

People respond to different things. You want to maintain that amount of professionalism so they see that it is a good service but this whole social media friendship, community thing, people relate to that now too, more than they used to.

HP12

#### Websites

Websites were seen to have greater depth of information than other platforms such as apps or text messages. The website concept was the most familiar mHealth element to most health professionals, whereas the majority of women saw the website as a back-up for alternate platforms including texting, apps, and social media.

It needs to be easy to access…but there’s so many websites and you have to login so much yeah passwords…whereas if it came up in your Facebook feed it’s just there. Or even just link to the website in a text, like it just took you straight there rather than, oh I need to remember to check that website once every week.

W8, focus group

#### Video Messages

Video messages were seen by both groups to aid visual learning, but there were concerns from two women that there may be an incurred cost. Two women commented that they would prefer reading to video messages.

I like the ideas of video messages.… Video messages can be so much more engaging. As long as they are not too long [to view on my phone].

W15, interview

#### Apps

No health professionals were aware of health promotion apps focused on pregnancy. Some women familiar with apps saw benefits with ease of access and provision of food, exercise, and weight tracking features. Two women commented that they had many apps that they never used.

I’d probably use an app. It would be good as a journal.

W8, focus group

I have so many apps on my phone, I never use them.

W5, focus group

#### Texts

Text messages were seen by all to be an avenue to communicate with women directly. Texting attributes noted included the ability to remind, motivate, and engage.

Using texts to reiterate that kind of information that you might have been told or might have actually read about but it will have gone out of your mind already.

W15, interview

One woman expressed concern that text messages intruded into other areas of life including paid work.

#### Social Networking or Forums

While social networks or forums were seen as a convenient way to create communities where common interests could be explored, some women were apprehensive that other women may be unsupportive or may make them feel anxious. The many opinions expressed on current social networks were seen to be overwhelming for some. The need for health professional moderation of social networks or forums was articulated by many. Some health professionals verbalized their fears of privacy breaches with social networks while others understood that if women were engaged in social networks, they had already accepted the privacy issues.

Forums are a good way to get the information across, and then people can make their own decision. You can check any time of the day. You can be involved as you want as well…

W2, focus group

I think there’s a lot of assumption that women are really supportive of other women. But I’m not sure that that’s actually the case when it comes to pregnancy and babies. There’s a lot of competitiveness and guilt.

W9, interview

### Optimizing User Engagement and Experience

Optimizing the user experience was seen to be crucial for an effective mHealth program. Both groups appealed for ease of access and use. Ideas to maximize engagement and motivation of women centered around tailoring and personalizing the intervention with messages concerning the baby’s development, content related to women’s interests, and presentation tailored to the technology platform. There appeared to be no ideal frequency of contact, with some women suggesting a preference of less than weekly contact and others preferring daily contact.

It might be, okay I’m not doing this for myself, I need to do this for someone else as well maybe.… It might just be a little bit extra of an influence, motivation to get out and do something.

W8, focus group

It’s good if you are tailoring it.… Because I would think that generic messages would be quite annoying if they’re not specific to you.

HP9

### Evidence-Based, Practical Content and Delivery

The need for continually updated, evidence-based information was voiced by both health professionals and women as fundamental to earn trust of women and health professionals. Some women and one health professional saw the “drip feeding” of information and engagement available with technology platforms as desirable in comparison to the chunking of information in print or oral forms. The suggestions for content were focused on the practical, including recipes, menus, and exercise plans.

[mHealth] should be shown to be based on up-to-date research…that you don’t have to question and think that they’re trying to sell me something or they’re anti-abortionists.

W9, interview

You hit all the key messages in the beginning…and they’re repeated at spaced-out intervals.

W7, focus group

There were differing views about who would be the best people to deliver information to women. Health professionals remarked that they had the credibility to deliver the information, which most women agreed with. Women reported mixed feelings about hearing from peers and their experiences. Listening to other women on social media was viewed by many to have created negative peer experiences for some, while others were happy to hear from peers if they were in a similar situation.

I think that the women want it from health professionals as opposed to peers because…otherwise she feels that she’d be judged.… I’m here as a health professional to help support them…and not judge them.

HP6

It’s nice to hear about other people’s experience, to put your mind at ease, but when you are taking advice, that’s a little bit different.

W8, focus group

## Discussion

### Principal Findings

This qualitative descriptive study aimed to explore women’s and health professionals’ views regarding a wide range of mHealth-based information sources and interventions promoting a healthy lifestyle in pregnancy. The study found that women held, in general, positive views of mHealth in promoting healthy nutrition, physical activity, and GWG in pregnancy. Health professionals’ views appeared more mixed and less positive overall, although some saw benefits of mHealth in antenatal care. The current study adds to the literature by highlighting stakeholder issues related to development, implementation, and evaluation of mHealth interventions and affirms the use of formative research as highlighted by others [19].

Variations in levels of interest for mHealth between health professionals and women were demonstrated in this study. Women consistently voiced enthusiasm for information technology integration in antenatal care and were able to describe development and implementation issues and information useful for inclusion. While some health professionals were positive regarding the potential for use of mHealth in antenatal care, others quickly identified perceived risks and barriers for implementation and were more likely to see mHealth as a service that would be used in parallel to face-to-face care. The diversity of health professionals’ attitudes in this study is consistent with other work considering professional views of mHealth in pregnancy [20] and primary health care interactions [34]. Although some health professionals regard technology as having the potential to improve patient knowledge and outcomes and enhance the patient-health professional relationship [34], Soltani and colleagues suggested that midwives were quick to identify limitations and risks due to these concerns being in line with their professional code of conduct of doing no harm [20]. In our study, lack of familiarity with technology, negative past interactions with women using technology, and fear of loss of information control also appeared to be associated with the concerns raised by health professionals. However, these are at odds with the finding that technology has the potential to improve patient knowledge and outcomes and enhance the patient-health professional relationship [34]. Further research is required to address health professionals’ concerns and teach the benefits of technology integration in antenatal care when developing models.

Women and health professionals in this study expressed the desire for multiple technology elements within an intervention as a way to broaden engagement and reach across various modes of technology and different learning styles. Further, it is likely that more than one element will be required to address the multiple components that together facilitate behavior change [29]. For example, websites may display large quantities of information most clearly, but social media or forums may provide a better avenue for peer support [35]. With mHealth still in its infancy, interventions have relied on using stand-alone technology platforms like text messaging and apps rather than using these features in combination with other opportunities afforded by mHealth. There has been a call for increasing the complexity of elements employed in mHealth interventions to fully exploit the functionality of portable devices and create more potent interventions [36,37]. Evidence suggests that greater engagement and improved health behavior outcomes may be facilitated by the utilization of more than one technology element [38]. It is acknowledged that increasing the complexity of mHealth interventions often increases financial, time, and resource costs. Over the last decade, new models for building and evaluating behavior change interventions have been proposed, with multiphase optimization strategy and sequential multiple assignment randomized trial designs offering promise [37]. Further research is required to understand the best combinations of technology elements to most efficiently elicit behavior change in different populations.

In this study, women expressed wide variability on acceptable rate of contact from an intervention, ranging from once per week to every day. For intervention developers, tension exists between communicating sufficiently to effect behavior change but not excessively to result in disengagement or adverse health outcomes such as stress. While more intensive interventions are associated with greater effect [39], this study and others [40] have found participants questioning the intrusiveness of intense interventions. This study supports the notion that tailoring interventions is crucial to suit women’s needs. It remains unclear as to how mHealth interventions can provide accurate and timely information and feedback without adverse effects on engagement [40]. Process and engagement evaluation from mHealth antenatal interventions will offer more insights in the future. A forerunner to this research is a (yet unpublished) process evaluation from the eMoms roc study [41]. The authors found differing engagement patterns by demographic and weight status subgroups. Future engagement evaluation coupled with outcome results has the potential to offer sophisticated insights for intervention targeting and development.

Peer or social support has been identified in weight-related interventions as central to successful health behavior change [29,42]. In the study, some women expressed concerns and cynicism about the helpfulness of peer support through groups, social media, or chat rooms and the role-modeling of behaviors by other women. The concerns appeared to be associated with negative social media experiences. Mixed findings have been reported in other research [43]. In a qualitative study with 35 overweight adults in the United States, real-time social or peer support through a virtual community was identified as a key benefit to mHealth interventions [43]. Conversely, a qualitative study with 19 students and staff at Southampton University in the United Kingdom demonstrated negative attitudes towards peer support in health promotion programs [40]. The authors of the UK study highlighted the need to investigate how to foster engaging and enjoyable social support environments in interventions.

### Strengths and Limitations

A strength of this study was the innovative approach including women and a wide range of health professionals to investigate opinions on mHealth in antenatal care. The broad range of those interviewed was unique and covered the range of health professionals who would see women from early pregnancy (community pharmacists) to birth (midwives and obstetricians). Conversely, having only two representatives for each health professional role may be viewed as a limitation. While construct saturation was reached, mHealth issues specifically related to their professions were not explored. Future research expanding and contrasting specific health professional views may be warranted.

The inclusion of focus group discussions and individual interviews with women utilized the positive aspects of each qualitative method. The interview-only methodology with health professionals was chosen for pragmatic reasons, and the lack of focus group interaction may have provided less in-depth, interactive, or rich information than was observed with the cohort of women [44]. The regional nature of sampling may place some limits on the generalizability of the outcomes. However, the similarity of findings overseas with midwives [20] suggests that many of the issues discussed may be commonplace.

###  Conclusions

This study found generally positive perceptions concerning mHealth for the promotion of healthy nutrition, physical activity, and GWG in antenatal care among women. Conversely, health professionals expressed a much wider variation in views and attitudes and were more able to identify potential risks and barriers to development and implementation. While most women could picture mHealth as an integral element in antenatal care and were able to identify variables required, health professionals were more likely to see mHealth as a parallel entity. These are unique data in the Australian context. In addition to improving the knowledge base, this research contributes to our understanding of the opportunities and challenges of developing mHealth interventions in antenatal care. Further, this study provides a foundation to inform the development of mHealth approaches that might be trialed in future studies.

## Multimedia Appendix 1

Questions and diagram used in focus groups and interviews.

## Abbreviations

GWG: gestational weight gain
