# Supplementary material for: Views of Women and Health Professionals on mHealth Lifestyle Interventions in Pregnancy: A Qualitative Investigation
Source: JMIR Mhealth Uhealth. 2015 Oct 28;3(4):e99. doi: 10.2196/mhealth.4869 (PMC4704935; doi:10.2196/mhealth.4869)
Supplement: Supplementary file 1 [file mhealth_v3i4e99_app1.pdf]

Additional file 1: Questions and diagram utilised in interviews and focus groups

Note that general questions regarding healthy diet, physical activity and GWG preceded these but are not included in this study.

1. Interview guide with health professionals

1. What do you think of delivering health education to pregnant women via mobile phones or the internet?

2. Do you think it is common for pregnant women to seek information via this manner?

Explanation of diagram (figure 1) as depicting multifaceted intervention elements to promote healthy nutrition, PA and GWG in pregnancy.

3. Could you see a model utilising the technology elements working for your patients? Do you think women would use it?

4. What elements appeal to you? Expand

5. What elements don't appeal to you? Expand

6. What do you think about having patient discussions on social media/chat forums?

7. How do you think a technology intervention would appeal to your patients?

8. How could you see it fitting into your clinic/pharmacy/practice?

2. Focus group/interview guide with women

1. How do you seek information about healthy lifestyles in pregnancy?

Explanation of diagram (figure 1) as depicting multifaceted intervention elements to promote healthy nutrition, PA and GWG in pregnancy.

2. Thinking about this program which elements appeal to you? Which do you think you would use? Which parts don't you like/wouldn't use?

3. Would you like to be part of this program during a pregnancy?

4. Thinking about contact from a program. How often do you think communication or texts giving information and motivation tips could be sent?

5. Thinking about the social media/chat forum element. How would you feel about discussing pregnancy lifestyle issues?

6. Women have told us that they would like to hear stories from real women. Who would you like to hear from?

7. If we included information and videos messages from health professionals which health professionals would you like to hear from and what type of messages?

8. How could we attract women to such a program?

Views of women and health professionals on mHealth lifestyle interventions in pregnancy: a qualitative investigation

Figure 1: Diagram of theoretical elements of a program promoting healthy nutrition, physical activity and weight during pregnancy
